# Extracts from the Minutes of the Atlanta Medical Society

**Published:** 1867-04

**Authors:** W. S. Armstrong

**Affiliations:** Demonstrator of Anatomy, Atlanta Medical College


### ARTICLE IV.

*Extracts from the Minutes of the Atlanta Medical Society.* By Dr. W. S. Armstrong, Demonstrator of Anatomy, Atlanta Medical College.

Dr. O'Keefe reported a case of Uterine hemorrhage, of three month's standing, in a lady about forty years old. She was the mother of several children; the youngest of which was eighteen months old. During the time stated, she had taken a number of remedies from another physician, with more or less benefit. The patient was feeble and auernic, had no appetite, pain in the back and illiac regions, and required the use of morphine to procure sleep. After the use of lead and opium, for about a week, an examination of the uterus, with speculum, was thought necessary.

No displacement or organic disease of that organ was discovered; but the os and cervix were pale, relaxed, and flabby. The cause of the hemorrhage was thought to be passive congestion, with a relaxed condition of the lining membrane of the uterus. The bromide of potassium was now administered in scruple doses, three times a day, with no benefit whatever. A combination of spirits ot turpentine, wine of ergot, tincture of cinnamon, and tincture of opium, was next employed, with the happiest effect. Under its use, in a few days, the hemorrhage gradually subsided, and has not returned since. Chalybeate tonics were given to restore the shattered health, which, in a few weeks, was fully established.

Dr. Alexander agreed with Dr. O'Keefe in the pathology of the case, and was in the habit of relying mainly on the spirits of turpentine in such cases.

Dr. W. F. Westmoreland mentioned a case of uterine hemorrhage recently seen, which had existed for a year or more, and which depended upon a soft intra-uterine tumor. The tumor was felt through the patulous os uteri, and was removed by drawing down the uterus with a hook, and separating it with the fingers.

Dr. J. M. Johnson had recently met with a case of constant uterine hemorrhage, in which he detected a solid substance in the os uteri, and finally extracted the result of a pregnancy. The hemorrhage, before and after this, was very annoying for several weeks. After the expulsion of the embryo, moles, or other similar organizations, escaped at different times,—hemorrhage continuing most of the time. At this stage, applications of cold water seemed to correct the hemorrhage. Ergot, he thought, should have been given when he first saw the case, but was not.

Dr. Alexander mentioned, in this connection, a case under his charge, at present five months advanced in pregnancy, in which frequent hemorrhage has occurred, without interfering with the development of the fœtus.

Dr. O'Keefe remarked that he had a case, in all respects, similar.

Dr. W. F. Westmoreland reported a case of delirium tremens, which terminated fatally. Covulsions had been present for a day or two, but subsided under the use of chloroform inhalations, and finally returned, and resulted in death.

Dr. Douglas thought chloroform useful in all cases of the disease.

Dr. Word mentioned a case of delirium tremens, with convulsions, occurring two or three weeks after hard drink, which was treated by opium, with no benefit. He became quiet under the use of chloroform inhalations; but the frequency of respiration was so seriously interfered with, as to require its suspension. Chloroform was then used internally, with the effect of increasing the delirium; and he died in that condition. He thought the remedy did an injury, and, perhaps, hastened the fatal issue.

Dr. Alexander reported a case of delivery, in which chloroform had been used, followed by excessive nausea. No hemorrhage appeared; but on turning the patient from one side to the other, a large amount of water was discharged, greatly to the relief of the patient. An explanation of this phenomenon having been asked by Dr. Alexander, some of the members expressed the opinion, that extra cysts existed in some instances of utero-gestation.

Dr. J. G. Westmoreland reported a case of painful condition of the anus, in its symptoms, resembling that of fissure. From digital examination nothing was discovered evcept internal hemorrhoids. He thought the pain unusual from this condition alone.

Dr. W. F. Westmoreland thought the pain resulted from violent contractiou of the sphyncter ani, probably from fissure.

Dr. Douglas thought that fissure imperfectly developed sometimes produced pain of this character.

Dr. Word said he agreed with Dr. W. F. Westmoreland in regarding this a case of fissure of the anus, and recommending the use of nitrate silver.

Dr. W. F. Westmoreland reported a case attacked with fever, violent pain in the head, and contractions of the fluxor muscles of the leg. He thought the symptoms were those of corebro-spinal meningitis. The patient died the third day. His object in mentioning the case, was to ellicit from members the plan of treatment pursued.

Dr. W. S. Armstrong said that he had met with many cases in the army. The most prominent symptoms were violent pain in the head, soreness and contraction of the muscles of the nape of the neck and back, often producing opisthotonos. Strabismus was occaionally met with; the pupils were sometime contracted, but most often dilated.

General hyperaesthesia and intollerance of light were present in many cases. The pulse was sometimes below the natural standard, but oftener above. These cases generally proved fatal in a very few days.

Post mortem examinations invariably revealed adventitious deposits of lymph of yellowish-green color between the membarnus of the brain, on the superior surface at the base, and around the spinal cord. Effusion of serum in the ventricles was present also.

He remarked, that he could not reccommend any particular plan of treatment as likely to prove beneficial. He has used quinine and opium, calomel, drastic cathartics, blisters to the head and spine, and cups; on the other hand, he has used stimulants, but with no better results.

Dr. W. F Westmorblend, in his treatment of the disease in the hospitals, did not find it so constantly fatal. He considered cerebro-spinal meningitis and diphtheria, only forms of malignant epidemic influenza.

Dr. J. M. Boring had found recovery to follow the use of large doses of calomel and opium, blisters to the nape of the neck, and cold to the head. He found the symptom of muscular contraction common in the cases observed by him.

Dr. W. F. Westmoreland mentioned the progress of the case mentioned at the last meeting, having symptoms of fissure of the anus. The case had been more critically examined, and fissure discovered, with painful contractions of the sphincter. Forcible extension of the sphincter, with the fingers, was resorted to, with the prospect of success.

